# Percutaneous Cryoablation under Conscious Sedation: A Safe, Effective and Painless Option for the Treatment of Pediatric Osteoid Osteoma

**DOI:** 10.3390/jcm12216889

**Published:** 2023-11-01

**Authors:** Claudio Pusceddu, Elva Vergantino, Domiziana Santucci, Salvatore Marsico, Matteo Cappucci, Federica Vaccarino, Bruno Beomonte Zobel, Rosario Francesco Grasso, Eliodoro Faiella

**Affiliations:** 1 Department of Oncological and Interventional Radiology, Businco Hospital, 09121 Cagliari, Italy; 2 Department of Radiology, Fondazione Policlinico Universitario Campus Bio-Medico, Via Alvaro del Portillo, 200, 00128 Roma, Italy; elva.vergantino@unicampus.it (E.V.); b.zobel@policlinicocampus.it (B.B.Z.); r.grasso@policlinicocampus.it (R.F.G.); e.faiella@policlinicocampus.it (E.F.); 3 Department of Radiology, Hospital del Mar, 08003 Barcelona, Spain; salvatore.marsico@hotmail.it; 4 Department of Interventional Radiology, Mater Olbia Hospital, 07026 Olbia, Italy

**Keywords:** osteoid osteoma, percutaneous treatment, cryoablation, pediatric patients, conscious sedation, interventional radiology

## Abstract

Background: Percutaneous CT-guided cryoablation is an emerging technique for treating bone tumors. However, experience with using this procedure for osteoid osteomas in pediatric patients remains limited. Our study aims to assess its technical feasibility, clinical efficacy, and safety in children treated under conscious anesthesia. Methods: We conducted a retrospective study of consecutive pediatric patients who underwent CT-guided percutaneous cryoablation for osteoid osteomas at our institution between September 2017 and March 2021. All patients received conscious anesthesia. Data on peri-procedural VAS scores, post-procedural VAS scores, imaging findings, and nonsteroidal anti-inflammatory drug (NSAID) usage rates were collected for each patient. Technical success was defined as proper cryoprobe placement at the nidus center, while clinical success referred to pain relief without NSAID use. Intra- and post-operative complications were also evaluated. Results: Nine patients underwent CT-guided percutaneous cryoablation for osteoid osteomas under conscious sedation, with a 100% overall success rate with low peri-procedural and median VAS scores (*p* < 0.01). No complications were observed during or after the procedure. Conclusions: CT-guided percutaneous cryoablation of pediatric osteoid osteomas is an effective and safe minimally invasive procedure feasible under conscious anesthesia, holding promise as a valuable treatment option.

## 1. Introduction

Osteoid osteoma ranks as the third most common benign bone-forming tumor, typically located in the cortex of long bones [1]. Approximately 70% of osteoid osteomas develop in patients younger than 20 years, with a male predominance of 2–3:1 [2]. These tumors exhibit limited growth potential and rarely exceed 1.5 cm in their largest dimension [3]. Patients afflicted with osteoid osteoma often experience severe pain, which starts off mild and intermittent, and may progress to more severe and persistent discomfort. Typically, the pain intensifies at night and responds to salicylates and NSAIDs [4].

**Figure 1:**
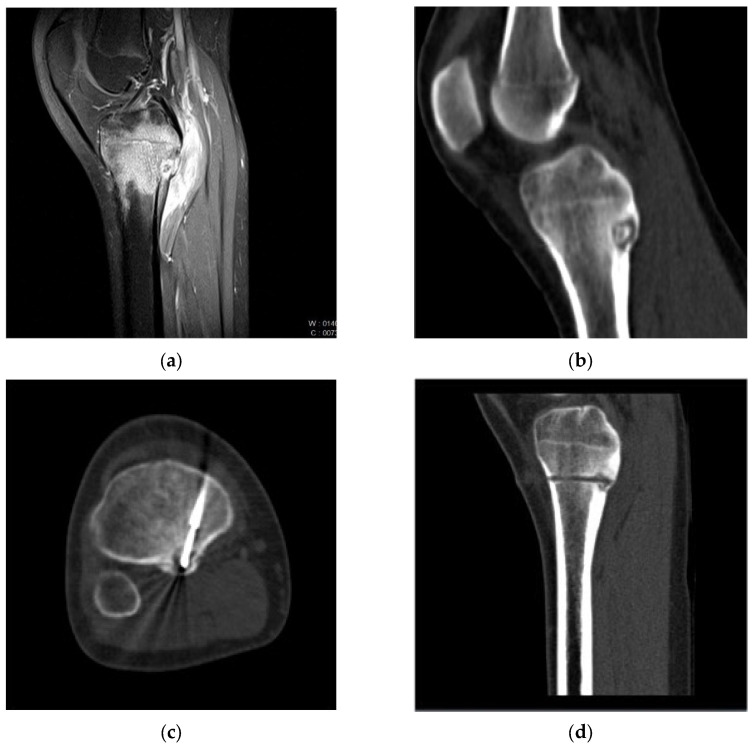
Pre-treatment CT (**a**) and MRI (**b**) images of a cortical Osteoid Osteoma in the posterior aspect of the tibia in a 14-year-old girl. (**c**) CT image during cryoablation: anterior access was performed to reach the “nidus” with the cryoprobe, aiming to avoid the neurovascular bundle. (**d**) Post-procedure CT image showing the trace of the Bonopty introducer needle path within which the cryoprobe is positioned.

Radiographically, osteoid osteomas present as well-circumscribed small masses consisting of a nidus, with or without central mineralization, surrounded by a zone of reactive sclerosis, cortical thickening, and bone marrow and soft tissue edema (Figure 1a,b) [5]. The tumor primarily manifests in the appendicular skeleton, with lower extremities more commonly affected than upper ones. Osteoid osteomas can be further classified as intracortical, subperiosteal, endosteal, or medullary, with the subperiosteal location in the diaphysis of the proximal femur and tibia being most prevalent (50% of cases) [6].

Long-term medical treatment is often poorly tolerated, and complete pain relief typically necessitates the destruction of the nidus. Emerging evidence suggests that percutaneous image-guided interventional techniques can replace surgical excision [7]. Presently, radiofrequency ablation (RFA) is the most commonly used technique, boasting a therapeutic success rate of 90% and a low complication rate [8,9,10,11]. However, the technical and clinical success rate of RFA is diminished in the pediatric population compared to adults, with a higher likelihood of therapeutic failure in children [12].

Image-guided cryoablation is a new percutaneous therapeutic option for patients with osteoid osteoma. Cryoablation involves the rapid freezing and thawing of target tissues using a percutaneously inserted cryoprobe, inducing changes in electrolyte and osmotic pressure that result in cell death [13].

Our study proposes an alternative method to radiofrequency for performing the ablative treatment of osteoid osteoma in a non-painful manner in the pediatric population. This suggests a technique that can be performed under conscious sedation rather than general anesthesia, which is a crucial aspect in this population.

Hence, our study’s primary objectives in pediatric osteoid osteoma cryoablation are to analyze technical success, assess the technique’s efficacy in pain resolution, and evaluate the procedure’s safety. Secondary objectives include peri-procedural pain analysis and post-procedure anti-inflammatory drug usage rates.

## 2. Materials and Methods

The study was conducted according to the guidelines of the Declaration of Helsinki. Ethical review and approval were waived for this study, due to the retrospective nature of the study.

### 2.1. Patients

All consecutive pediatric patients treated with CT-guided percutaneous cryoablation between September 2017 and March 2021 were included. Inclusion criteria were as follows: clinical presentation of osteoid osteoma with a pain score > 4 on the Visual Analog Scale (VAS) (0–10), persisting for at least 6 months, worsening at night, and responsive to salicylates and NSAIDs; CT/MR diagnosis of osteoid osteoma; patients under 18 years of age; and the availability of clinical and radiological follow-up data, including post-procedure MRI at approximately 6 months post-cryoablation. Four patients were excluded due to missing imaging and clinical follow-up data.

**Figure 2:**
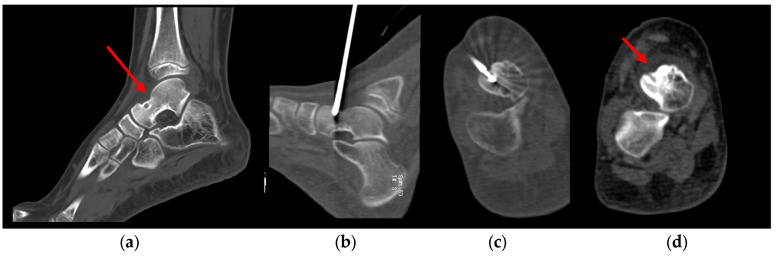
Pre-treatment CT image (**a**) of an osteoid osteoma in the talus in a 12 years old child with a persistent limp for 8 months; (**b**) sagittal and (**c**) axial CT image during cryoablation procedure using an IceSeed cryoprobe; (**d**) 1-month post-procedure CT image.

A total of 9 patients were identified (3 females and 6 males, 7–16 years, median age of 12.8 years). Tumor locations included the tibia with cortical lesions (n = 3), femur with cortical lesions (n = 2), astragalus with intraspongiosa lesions (n = 2) (Figure 2), ilium with intraspongiosa lesions (n = 1), and one spinal osteoid osteoma located in the intraspongiosa site of D12 (n = 1).

### 2.2. Procedure

Two specialized and experienced musculoskeletal interventional radiologists conducted the procedures. Parental/tutor informed consent was obtained, and patients were informed about potential complications. Before the procedure, local anesthesia (1% Lidocaine) was administered at the skin entry site and periosteum, and all patients underwent conscious sedation (Midazolam and Tramadol). Procedures were performed using a multidetector CT (SOMATOM go.Top 128, SIEMENS Healthineers, Erlangen, Germany). Initially, the lesion’s morphological characteristics were identified through a non-contrast CT scan. For each procedure, access to the osteoid osteoma was established using a Bonopty introducer needle 14G (Apriomed, Derry, NH, USA), equipped with a manual drill and a biopsy needle, enabling the collection of a pathological tissue sample for histological diagnosis of osteoid osteoma.

Subsequently, cryoablation was performed using a cryoablation system (VISUAL ICE, Galil Medical-Boston Scientific, Arden Hills, MN, USA) equipped with a single 17G insulated cryoprobe capable of generating ablation zones with different diameters: IceSphere 1.5 (22 × 28 mm at −20 °C) and IceSeed 1.5 (15 × 20 mm at −20 °C). Most cryoprobes used were IceSeed cryoprobes (n = 7), while IceSphere cryoprobes were employed in two patients (n = 2), with a reduced freezing cycle power (50% of max power).

The cryoprobe was inserted coaxially through the Bonopty introducer, positioning its extremity at the nidus’s center (Figure 1d). Intra-procedural scans were performed to confirm the instrument’s position and direction. A single 10 min freezing cycle followed by an 8 min passive thaw cycle was executed and non-contrast CT images were obtained at the end of the freezing phase to assess the extent of the iceball and detect immediate complications (Figure 1c). The VAS score was collected immediately after the procedure, within 1 h of the procedure (peri-procedural VAS score). Patients were monitored during the first 48 h and then discharged two days after the procedure if there was no discomfort.

### 2.3. Follow-Up

All patients were reevaluated at the interventional radiology department, recording pain VAS scores at 1 week, 1 month, and 3 months post-procedure. Additionally, an MRI was conducted 1-month post-procedure to investigate potential complications (stress reaction/fracture, osteonecrosis, joint effusion/synovitis, articular cartilage damage, adjacent soft tissue collections or injury), and to assess the ablation zone.

### 2.4. Outcome Definitions

**Figure 3:**
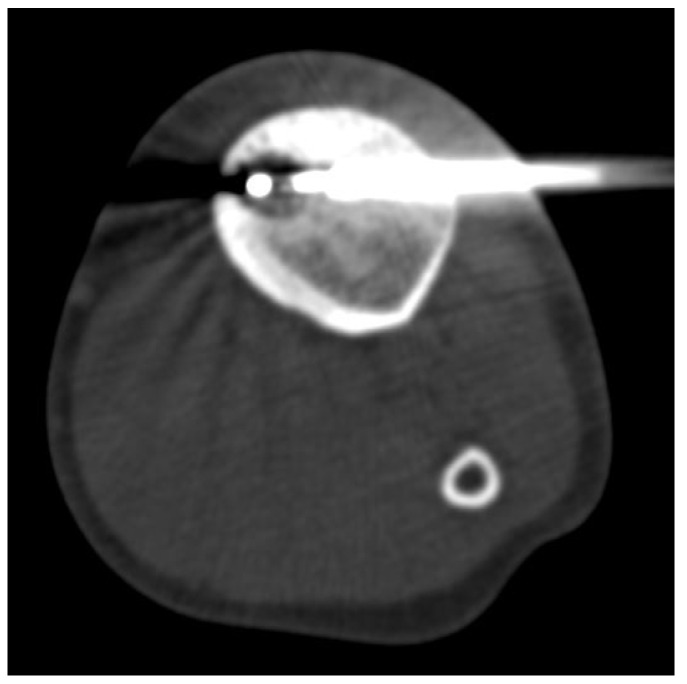
Cryoablation of tibial Osteoid Osteoma. CT image during the procedure showing the extent of the iceball’s extent and the distal tip of the cryoprobe at the center of the “nidus”. In this procedure, we performed a contralateral access to the “nidus” to avoid the thickened cortical portion.

Technical success was defined as the ability to place the cryoprobe’s extremity at the nidus’s center under CT guidance (Figure 3), with ablation performed for the desired period and desired coverage, accompanied by low-grade intraprocedural pain. Clinical success referred to a significant reduction in pain after 1 week and complete pain relief after 1 month and 3 months, without the need for salicylates or NSAIDs, enabling full return to daily activities and sports.

## 3. Results

Statistical analysis were performed using SPSS software (v.22). Statistical analysis of VAS scores before and after the procedure was performed using a paired *t*-Test to determine if post procedure VAS scores were statistically lower than pre-procedure VAS scores (1 week, 1 month and 3 months). *p* values less than 0.05 were considered statistically significant.

Technical success was achieved in all patients (n = 9) undergoing percutaneous cryoablation for OOs. The median duration of the cryoablation procedure was 18 min (range 13–23), and the median duration of the entire procedure was 60 min (range 45–90). Within 1 h of the procedure, two patients reported VAS pain scores of 0 (n = 2), three patients reported scores of 1 (n = 3), and four patients reported scores of 2 (n = 4), resulting in a mean peri-procedural VAS score of 1.2. All patients (n = 9) were discharged home two days after the procedure without post-operative complications. The overall clinical success rate was 100%. The median pre-procedure VAS score was 5.55, while the median post-procedure VAS score was 1.22 at the 1-week follow-up, demonstrating a statistically significant difference (*p* < 0.01) between pre-procedure and 1-week post-procedural VAS scores. At subsequent follow-ups (1 month and 3 months), all patients (n = 9) reported a VAS pain score of 0. None of the patients used anti-inflammatory drugs from the day after the procedure, although all had used them before treatment. No delayed complications were observed during the follow-up period.

## 4. Discussion

Our retrospective study aims to assess the technical and clinical success, specifically in terms of pain resolution, and safety of percutaneous cryoablation for osteoid osteomas in children and adolescents. In our study involving nine pediatric patients, we achieved a 100% success rate both clinically and technically, with no post-procedural complications and a significant reduction in pain just after the procedures, remaining still low at one week and during follow-ups after the procedure.

Since its introduction by Rosenthal in 1992, RFA treatment of osteoid osteomas has largely replaced surgical excision, leading to reduced hospitalization costs, shorter duration, and a low rate of complications and recurrence [6]. However, the success rate in pediatric patients is slightly lower than that in adults. Ragab et al. reported a technical success rate of 91.3% in 23 patients, with one case of failure due to excessive sclerosis hindering the correct positioning of the RF thermal ablation probe within the nidus, and another case involving hyperthermia that reduced the ablation time to 2 min [14]. Vanderschueren et al. observed that patients with an average age of 24 years had a lower risk of treatment failure with RFA for osteoid osteoma compared to those with an average age of 20 years [15].

Cryoablation is emerging as a promising percutaneous therapeutic option for patients with osteoid osteoma [16]. This technique offers several advantages: real-time monitoring of cryoprobe positions and cryosphere evolution using non-contrast-enhancement CT scans, the ability to use multiple cryoprobes simultaneously for tailoring the iceball to the tumor’s size and morphology and intrinsic anesthetic properties of technique that reduce peri- and post-procedural pain compared to RF ablation. Moreover, cryoprobe-induced ice balls can easily penetrate the cortex allowing for the formation of ice balls within the bone by placing in an extraosseous position. This results in decreased intraoperative pain, reduced requirements for analgesics, and preservation of the extracellular matrix [17]

This makes cryoablation well-tolerated under conscious sedation [18]. Several studies have shown that patients undergoing cryoablation do not experience increased periprocedural pain as seen in radiofrequency ablation patients [19,20].

While cryoablation of OOs has been demonstrated to be technically feasible with high success rates in adults [21], limited studies have analyzed its application in pediatric populations. Wu et al. achieved technical and clinical success in all six pediatric patients treated with cryoablation, with no major complications [12]. Liu et al. examined two pediatric case reports on cryoablation treatment of OOs, reporting technical success [22]. Whitmore et al. demonstrated that cryoablation is a technically feasible (100% technical success) and clinically effective (90.5% long-term clinical success) therapeutic option for a population of 29 children [13].

Furthermore, Le Coroller et al. have demonstrated the efficacy of cryoablation even for intra-articular lesions in adults and it could potentially be a better treatment option for the treatment of intra-articular lesions in pediatric patients, particularly considering the heightened vulnerability to cartilage damage. However, comparative studies with radiofrequency ablation (RFA) remain unavailable [21].

Technical and clinical success in cryoablation are interrelated, with clinical success hinging on technical success and the correct positioning of the cryoprobe. In our study, all procedures were successful, achieving 100% clinical success by placing the cryoprobe’s distal end at the nidus’s center.

In addition to demonstrating the technical and clinical success of cryoablation in the pediatric population, our study highlights the feasibility of performing cryoablation under conscious anesthesia. Peri-procedural VAS score (within 1 h) was assessed, with all patients reporting pain equal to or less than 2 (mean peri-procedural VAS score 1.2), indicating that the procedure can be tolerated without the need for general anesthesia.

The treatment involves the use of a single cryoprobe with a cost slightly higher than the RFA electrode, however, contained within the procedural reimbursement. In both cases follow up includes 1-month MRI ad clinical evaluation of pain resolution. However, the benefits of regional anesthesia with conscious sedation in contrast to general anesthesia lead to decreased anesthesia-linked side effects, shorter procedural time, reduced care unit staying, and accelerated recovery with faster hospital discharge [23].

This not only enabled a reduction in anesthetic quantities but also facilitated the transition from high-potency analgesics like Fentanyl to low-potency analgesics such as Tramadol. This represents a crucial facet especially in the pediatric population.

In clinical practice, management of osteoid osteoma can be based on clinical presentation and imaging without the need for histological diagnosis. However, one limitation of previous studies is the lack of systematic histologic tumor verification [13,21]. In our study, we performed lesion biopsies in all patients, confirming the histological diagnosis of osteoid osteoma in each case.

There are two main potential disadvantages of this technique that should be discussed. First, the duration of the procedure. In general, the duration of the cryoablation procedure is on average longer than that of RF, but in our institute the entire cryoablation procedure requires about 60 min, which is less than the entire RF duration reported by some studies (90–120) [24,25] and therefore shorter than the cryoablation procedures described in the literature [12,21]. This can be explained by the fact that our patients underwent conscious anesthesia, which requires less time than general anesthesia. In addition, due to the benign nature of the tumor, compared to previous studies in which on average more than one freeze–thaw cycle was performed [13,21,22], a single freeze–thaw cycle (10 min freezing cycle followed by 8- minutes passive thaw cycle) was performed in all of our procedures, resulting in a shorter effective duration of cryoablation. Second, one of the complications associated with cryoablation is the appearance of skin injuries (skin blisters) [13]; to prevent this, isolated cryoprobes were used in our study, avoiding placing a sterile glove filled with warm saline around the needle during the freezing cycle. There are some main limitations in our study, including the small number of patients, the lack of a 12-month follow-up and the high costs of the procedure. In addition, only two of the treated patients were younger than 10 years (ages 7 and 8 years), so our results cannot be fully extended to a younger population.

The future developments of the study should involve assessing the outcomes of the technique applied to a larger patient cohort, based on a more extended follow-up period and a randomized comparative study of the clinical efficacy compared to radiofrequency ablation.

## 5. Conclusions

CT-guided percutaneous cryoablation of pediatric osteoid osteomas is an effective, safe and painless minimally invasive procedure in our experience, being feasible under conscious sedation and resulting a valid option to RFA for pediatric patients.

## Data Availability

Data are available on request.

## References

[1] Zhang Y., Rosenberg A.E. (2017). Bone-Forming Tumors. Surg. Pathol. Clin..

[2] Tepelenis K., Skandalakis G.P., Papathanakos G., Kefala M.A., Kitsouli A., Barbouti A., Tepelenis N., Varvarousis D., Vlachos K., Kanavaros P. (2021). Osteoid Osteoma: An Updated Review of Epidemiology, Pathogenesis, Clinical Presentation, Radiological Features, and Treatment Option. In Vivo.

[3] Yalcinkaya U., Doganavsargil B., Sezak M., Kececi B., Argin M., Basdemir G., Oztop F. (2014). Clinical and morphological characteristics of osteoid osteoma and osteoblastoma: A retrospective single- center analysis of 204 patients. Ann. Diagn. Pathol..

[4] Malghem J., Lecouvet F., Kirchgesner T., Acid S., Vande Berg B. (2020). Osteoid osteoma of the hip: Imaging features. Skelet. Radiol..

[5] Chai J.W., Hong S.H., Choi J.Y., Koh Y.H., Lee J.W., Choi J.A., Kang H.S. (2010). Radiologic diagnosis of osteoid osteoma: From simple to challenging findings. Radiographics.

[6] De Filippo M., Russo U., Papapietro V.R., Ceccarelli F., Pogliacomi F., Vaienti E., Piccolo C., Capasso R., Sica A., Cioce F. (2018). Radiofrequency ablation of osteoid osteoma. Acta Bio Medica.

[7] Rosenthal D., Callstrom M.R. (2012). Critical review and state of the art in interventional oncology: Benign and metastatic disease involving bone. Radiology.

[8] Rosenthal D.I., Alexander A., Rosenberg A.E., Springfield D. (1992). Ablation of osteoid osteomas with a percutaneously placed electrode: A new procedure. Radiology.

[9] Gangi A., Alizadeh H., Wong L., Buy X., Dietemann J.L., Roy C. (2007). Osteoid osteoma: Percutaneous laser ablation and follow-up in 114 patients. Radiology.

[10] Rimondi E., Mavrogenis A.F., Rossi G., Ciminari R., Malaguti C., Tranfaglia C., Vanel D., Ruggieri P. (2012). Radiofrequency ablation for non-spinal osteoid osteomas in 557 patients. Eur. Radiol..

[11] Lanza E., Thouvenin Y., Viala P., Sconfienza L.M., Poretti D., Cornalba G., Sardanelli F., Cyteval C. (2014). Osteoid osteoma treated by percutaneous thermal ablation: When do we fail? A systematic review and guideline for future reporting. Cardiovasc. Interv. Radiol..

[12] Wu B., Xiao Y.Y., Zhang X., Zhao L., Carrino J.A. (2011). CT-guided percutaneous cryoablation of osteoid osteoma in children: An initial study. Skelet. Radiol..

[13] Whitmore M.J., Hawkins C.M., Prologo J.D., Marshall K.W., Fabregas J.A., Yim D.B., Monson D., Oskouei S.V., Fletcher N.D., Williams R.S. (2016). Cryoablation of Osteoid Osteoma in the Pediatric and Adolescent Population. J. Vasc. Interv. Radiol..

[14] Donkol R.H., Al-Nammi A., Moghazi K. (2008). Efficacy of percutaneous radiofrequency ablation of osteoid osteoma in children. Pediatr. Radiol..

[15] Vanderschueren G.M., Taminiau A.H., Obermann W.R., van den Berg-Huysmans A.A., Bloem J.L. (2004). Osteoid osteoma: Factors for increased risk of unsuccessful thermal coagulation. Radiology.

[16] Cazzato R.L., Garnon J., Ramamurthy N., Koch G., Tsoumakidou G., Caudrelier J., Arrigoni F., Zugaro L., Barile A., Masciocchi C. (2016). Percutaneous image-guided cryoablation: Current applications and results in the oncologic field. Med. Oncol..

[17] Coupal T.M., Mallinson P.I., Munk P.L., Liu D., Clarkson P., Ouellette H. (2014). CT-guided percutaneous cryoablation for osteoid osteoma: Initial experience in adults. Am. J. Roentgenol..

[18] Thacker P.G., Callstrom M.R., Curry T.B., Mandrekar J.N., Atwell T.D., Goetz M.P., Rubin J. (2011). Palliation of painful metastatic disease involving bone with imaging-guided treatment: Comparison of patients’ immediate response to radiofrequency ablation and cryoablation. Am. J. Roentgenol..

[19] Callstrom M.R., Kurup A.N. (2009). Percutaneous ablation for bone and soft tissue metastases—Why cryoablation?. Skelet. Radiol..

[20] Moser T., Buy X., Goyault G., Tok C., Irani F., Gangi A. (2008). Image-guided ablation of bone tumors: Review of current techniques. J. Radiol..

[21] Le Corroller T., Vives T., Mattei J.C., Pauly V., Guenoun D., Rochwerger A., Champsaur P. (2022). Osteoid Osteoma: Percutaneous CT-guided Cryoablation Is a Safe, Effective, and Durable Treatment Option in Adults. Radiology.

[22] Liu D.M., Kee S.T., Loh C.T., McWilliams J., Ho S.G., Brower J.S., Munk P.L. (2010). Cryoablation of osteoid osteoma: Two case reports. J. Vasc. Interv. Radiol..

[23] Vanderschueren G.M., Taminiau A.H., Obermann W.R., Bloem J.L. (2002). Osteoid osteoma: Clinical results with thermocoagulation. Radiology.

[24] Rosenthal D.I., Hornicek F.J., Torriani M., Gebhardt M.C., Mankin H.J. (2003). Osteoid osteoma: Percutaneous treatment with radiofrequency energy. Radiology.

[25] Santiago E., Pauly V., Brun G., Guenoun D., Champsaur P., Le Corroller T. (2018). Percutaneous cryoablation for the treatment of osteoid osteoma in the adult population. Eur. Radiol..

